# A Straightforward Assay for Screening and Quantification of Biosurfactants in Microbial Culture Supernatants

**DOI:** 10.3389/fbioe.2020.00958

**Published:** 2020-08-20

**Authors:** Sonja Kubicki, Isabel Bator, Silke Jankowski, Kerstin Schipper, Till Tiso, Michael Feldbrügge, Lars M. Blank, Stephan Thies, Karl-Erich Jaeger

**Affiliations:** ^1^Institute of Molecular Enzyme Technology, Heinrich Heine University Düsseldorf, Jülich, Germany; ^2^Forschungszentrum Jülich GmbH, Bioeconomy Science Center (BioSC), Jülich, Germany; ^3^iAMB-Institute of Applied Microbiology, ABBt-Aachen Biology and Biotechnology, RWTH Aachen University, Aachen, Germany; ^4^Center of Excellence on Plant Sciences, Institute for Microbiology, Heinrich Heine University Düsseldorf, Düsseldorf, Germany; ^5^Forschungszentrum Jülich GmbH, Institute of Bio- and Geosciences IBG 1: Biotechnology, Jülich, Germany

**Keywords:** biosurfactants, colorimetric assay, screening, quantification, rhamnolipid, recombinant production, Victoria Pure Blue BO

## Abstract

A large variety of microorganisms produces biosurfactants with the potential for a number of diverse industrial applications. To identify suitable wild-type or engineered production strains, efficient screening methods are needed, allowing for rapid and reliable quantification of biosurfactants in multiple cultures, preferably at high throughput. To this end, we have established a novel and sensitive assay for the quantification of biosurfactants based on the dye Victoria Pure Blue BO (VPBO). The assay allows the colorimetric assessment of biosurfactants directly in culture supernatants and does not require extraction or concentration procedures. Working ranges were determined for precise quantification of different rhamnolipid biosurfactants; titers in culture supernatants of recombinant *Pseudomonas putida* KT2440 calculated by this assay were confirmed to be the same ranges detected by independent high-performance liquid chromatography (HPLC)-charged aerosol detector (CAD) analyses. The assay was successfully applied for detection of chemically different anionic or non-ionic biosurfactants including mono- and di-rhamnolipids (glycolipids), mannosylerythritol lipids (MELs, glycolipids), 3-(3-hydroxyalkanoyloxy) alkanoic acids (fatty acid conjugates), serrawettin W1 (lipopeptide), and *N-*acyltyrosine (lipoamino acid). In summary, the VPBO assay offers a broad range of applications including the comparative evaluation of different cultivation conditions and high-throughput screening of biosurfactant-producing microbial strains.

## Introduction

Microbial biosurfactants are a structurally heterogeneous group of secondary metabolites. Surface active natural products are for instance glycolipids like rhamnolipids, trehalolipids, sophorolipids, and mannosylerythritol lipids (MELs) as well as lipopeptides (or lipoamino acids like) surfactin, serrawettin W1, *N*-acylamino acids, or polymers like emulsan (Soberón-Chávez et al., 2011; Jahan et al., 2020). Microbial biosurfactants are gaining increasing attention as an alternative to petrol-based surfactants and are one of many natural compounds which can contribute to a biobased economy (Olasanmi and Thring, 2018; Naughton et al., 2019). Several biosurfactants already reached the level of industrial production including rhamnolipids, sophorolipids, MELs, and surfactin (Erum et al., 2013; Singh et al., 2019). The best-known producer of rhamnolipids is the bacterial pathogen *Pseudomonas aeruginosa*; sophorolipids and MELs are produced with high titers by different yeasts. These glycolipids are discussed for many applications, e.g., in cosmetics, household detergents, or environmental remediation (Khan et al., 2014). Additionally, many lipopeptides are well-known for their pronounced bioactivities; an example is serrawettin W1 which is naturally produced by the pathogenic bacterium *Serratia marcescens* DSM12481 (Gudiña et al., 2016; Hage-Hülsmann et al., 2018).

Recent advances in synthetic biology and biotechnology offer new possibilities to fully capture the metabolic potential of microorganisms for biosurfactant production and thus increase their economic competitiveness (Perfumo et al., 2010; Geys et al., 2014). Heterologous and engineered non-pathogenic production strains give access to chemically diverse and tailored compounds and enable implementation of more cost-effective processes (Kubicki et al., 2019). In this context, *Pseudomonas putida* KT2440 proved to be especially promising for the production of bacterial biosurfactants like rhamnolipids (Wittgens et al., 2011; Loeschcke and Thies, 2020).

Simple and robust high-throughput identification methods complemented by high-resolution but time-consuming quantification methods like high-performance liquid chromatography (HPLC) and techniques such as mass spectrometry are needed to develop new strategies and identify production strains (Chen et al., 2007). Several methods have been described for the detection of rhamnolipids (Varjani and Upasani, 2017). These techniques visualize the surfactant-induced change in surface tension, examples include the drop collapse-, oil spreading-, grid-, or atomized oil-assay, or rely on hemolytic activity, complex formation with of pigments or fluorophores, or detection reactions with sugar components (Youssef et al., 2004; Burch et al., 2010; Laabei et al., 2014; Yang et al., 2015; Varjani and Upasani, 2017). These methods typically lack specificity for chemically diverse biosurfactants and, hence, are prone to interference with chemical and biological compounds in culture media, demand time-consuming extraction procedures, or do not deliver quantitative information. These features restrict the application of standard assays for reliable high-throughput screening of large sample numbers. Currently, the field of biosurfactant biology and biotechnology is rife with mal-reported compounds and their alleged production from various microbes, with many reports often lacking the rigor required to identify and quantify the surface-active compound under study (Irorere et al., 2017). In addition, a robust and reliable method to determine residual levels of cationic and non-ionic detergents (CTAB, C12E8, Tween 80) in vaccine preparations under defined conditions was described (Roosloot and Schoen, 2011). The method is based on the detergent-dependent solubilization of a dye used commercially in ball-pen inks (Miller et al., 1958). This assay was reported to allow the determination of the critical micelle concentration (CMC) of defined detergents without tensiometric equipment (Vulliez-Le Normand and Eisele, 1993; Little et al., 1994; Chandran and Nibert, 1998).

These studies prompted us to develop a robust and sensitive colorimetric assay for the detection and quantification of biosurfactants in culture supernatants using the dye Victoria Pure Blue BO (established acronyms VPBBO or VPBO). We established a protocol for determination of biosurfactant concentrations in microtiter plates (MTPs) and evaluated the assay with the purified biosurfactants 3-(3-hydroxyalkanoyloxy) alkanoic acid (HAA), mono-rhamnolipids (mRL), and di-rhamnolipids (dRL). The assay was used for the quantification of rhamnolipid biosurfactants in recombinant *P. putida* KT2440 culture supernatants. Furthermore, we have demonstrated the suitability of the VPBO assay to detect chemically different biosurfactants in culture supernatants, namely, the non-ionic glycolipid MEL produced by *Ustilago maydis* MB215, the lipopeptide serrawettin W1 produced by recombinant *P. putida* KT2440, and *N*-acyltyrosines in culture supernatants from *Escherichia coli* DH10b.

## Materials and Methods

### Strains, Plasmids, and Culture Conditions

All strains used in this study are listed in Table 1. Bacterial strains were cultivated for 6 and 24 h after inoculation from a preculture in LB medium (10 g/L tryptone, 5 g/L yeast extract, 10 g/L NaCl, Carl Roth) to an optical density of OD_580nm_ = 0.05 in 1 ml LB medium containing 10 g/L glucose in sterile 48-well flowerplates (MTP-48-OFF, m2p-labs GmbH, Aachen, Germany) using a deep-well plate incubator (ThermoMixer C, Eppendorf, Hamburg, Germany, 1,200 rpm). *P. putida* KT2440 harboring pVLT plasmids was grown in the presence of 25 mg/L kanamycin for plasmid maintenance. Expression of *swrW* was induced by adding isopropyl-β-D-thiogalactopyranoside (IPTG; 0–2 mM) to the culture after 3.5 h of growth. Cultures of *E. coli* DH10b containing pEBP plasmids were supplemented with 50 mg/L kanamycin. *P. putida* KT2440 cultures were grown with orbital shaking at 30°C, *E. coli* DH10b cultures at 37°C. Culture supernatants were prepared by centrifugation in a tabletop centrifuge (1 min, 18,000 × g). All cultivations for validation of the VPBO assay were conducted as biological triplicates. The pH of the supernatants was determined using a Laboratory pH Meter 766 Calimatic (Knick GmbH & Co. KG, Berlin, Germany).

**TABLE 1:** Strains used in this study.

**Strains and plasmids**	**Characteristics**	**References or sources**
*E. coli* DH10b	*F^–^ mcrA Δ (mrr-hsdRMS-mcrBC) Φ80lacZ ΔM15 ΔlacX74 endA1 recA1 deo^*R*^ Δ(ara, leu)7697 araD139 galU galK nupG rpsLλ^–^*	Durfee et al., 2008
*P. putida* KT2440	Wildtype	Bagdasarian et al., 1981
*E. coli* DH10b pEBP18	*E. coli* DH10b harboring plasmid pEBP18 (ColE1 ori, *Km*^*R*^, *Cm*^*R*^, GFPmut3, P*_*T7*_*, P*_*xyl*_*, *cosNBQ*, BS*_amyEupdw)*	Thies et al., 2016
*E. coli* DH10b pEBP18_BA354	*E. coli* DH10b harboring plasmid pEBP18_BA354 (ColE1 ori, *Km*^*R*^, *Cm*^*R*^, GFPmut3, META*_nas354_pep_gly_oxi_ABC-tra*, P*_*T7*_*, P*_*xyl*_*, *cosNBQ*, BS*_amyEupdw)*	Thies et al., 2016
*P. putida* KT2440 pSB01	*P. putida* KT2440 harboring plasmid pSB01 (ColE1 ori, oriT, *Km*^*R*^, P*_*syn8*_*_PA_*rhlA*)	Tiso et al., 2017
*P. putida* KT2440 pPS05	*P. putida* KT2440 harboring plasmid pPS05 (pBBR1 ori, oriT, *Tc*^*R*^, *Km*^*R*^, P*_*syn16*_*_PA_*rhlAB*)	Tiso et al., 2016
*P. putida* KT2440 pWJ02	*P. putida* KT2440 harboring plasmid pWJ02 (pBBR1 ori, oriT, *Tc*^*R*^, P*_*syn16*_*_PA_*rhlABC*)	Tiso et al., 2017
*P. putida* KT2440 pVLT33	*P. putida* KT2440 harboring plasmid pVLT33 (pRSF1010 ori, oriT, *Km*^*R*^, EC_*lacI*, P*_*tac*_*)	Hage-Hülsmann et al., 2018
*P. putida* KT2440 pVLT-s*wrW*	*P. putida* KT2440 harboring plasmid pVLT33 (pRSF1010 ori, oriT, *Km*^*R*^, EC_*lacI*, P*_*syn16*_*_SM_*swrW*)	Hage-Hülsmann et al., 2018
*U. maydis* MB215	Wild type	Hewald et al., 2005
*U. maydis* MB215 Δrua1	Δ*rua1_HygR*	Hewald et al., 2005
*U. maydis* MB215 Δemt1	Δ*emt1_HygR*	Hewald et al., 2005
*U. maydis* MB215 Δcyp1Δemt1	Δ*cyp1*Δ*emt1_HygR*	Teichmann et al., 2010

*U. maydis* MB215 strains were cultivated as described before (Müller et al., 2018) in shake flasks at 28°C for 20 h. All cultivations were performed in modified Verduyn medium (10 g/L glucose, 0.9 g/L NH_4_NO_3_, 0.5 g/L KH_2_PO_4_, 0.2 g/L MgSO_4_ × 7 H_2_O, 0.01 g/L FeCl_3_ × 6 H_2_O and 1 ml/L trace element solution). The trace element solution contains 15 g/L ethylenediaminetetraacetic acid (EDTA), 4.5 g/L ZnSO_4_ × 7 H_2_O, 1.3 g/L MnCl_2_ × 6 H_2_O, 0.3 g/L CoCl_2_ × 6 H_2_O, 0.3 g/L CuSO_4_ × 5 H_2_O, 0.4 g/L Na_2_MoO_4_ × 2 H_2_O, 4.5 g/L CaCl_2_ × 2 H_2_O, 1 g/L B(OH)_3_, 0.1 g/L KI. The medium was buffered with 0.1 g/L 2-(N-morpholino)ethanesulfonic acid (MES, pH 6.5). Culture supernatants were harvested by centrifugation (15 min, 18,800 × g). Cultivations tested with the VPBO assay were grown as biological duplicates.

### Isolation and Thin-Layer Chromatography of Biosurfactants

Samples containing *N*-myristoyltyrosine or serrawettin W1 were prepared by extracting 500 μl culture supernatant from recombinant *E. coli* DH10b and *P. putida* KT2440 obtained by centrifugation (1 min, 18,000 × g) with 3 × 500 μl of ethyl acetate. After vortexing, the separation of the organic phase was performed by centrifugation in a tabletop centrifuge (30 s, 18,000 × g). The organic phases of the three extractions were collected, and the solvent was evaporated in a vacuum centrifuge (Speed-Vac concentrator 5301, Eppendorf, 120 min, 45°C). The solid pellet was dissolved in 20 μl ethanol, and 10 μl were separated by thin-layer chromatography (TLC) on silica 60 plates (SIL-G, Macherey-Nagel, Düren, Germany). Chemically synthesized *N*-myristoyltyrosine (Thies et al., 2016) and a chloroform extract of *S. marcescens* DSM12481 grown on solid tryptone-glycerol medium (Thies et al., 2014), respectively, were used as references. To distinguish between serrawettin W1 and prodigiosin in this extract, ethanol extracts of heterologously produced prodigiosin (Domröse et al., 2015) were additionally spotted on the TLC plates. TLCs were developed as described before using a mixture of chloroform, methanol, and acetic acid (65:15:2, v/v/v) for *N*-myristoyltyrosine and a mixture of chloroform, methanol, and 7 M ammonia in methanol (85:26:3, v/v/v) for serrawettin W1 as mobile phase. For visualization of *N*-myristoyltyrosine and serrawettin W1, the TLC plates were stained after solvent evaporation by exposure to iodine vapor from sublimating crystals in a beaker for 30 min.

For TLC with glycolipids from *U. maydis* MB215, supernatants were extracted by mixing 500 μl culture broth and 500 μl of ethyl acetate. The ethyl acetate phase was collected and evaporated, and glycolipids were dissolved in 15 μl methanol. Extracts were analyzed by TLC on silica plates (Silica gel 60; Merck) with a three-step solvent system. The first step included 5-min incubation in a buffer consisting of chloroform, methanol, and water (65:25:4, v/v/v), followed by 2 × 17 min in chloroform and methanol (9:1, v/v). Sugar-containing compounds were visualized by spraying with a mixture of acetic acid, sulfuric acid, and *p*-anisaldehyde (50:1:0.5, v/v) and heating at 110°C for 5 min.

### Analysis and Purification of 3-(3-Hydroxyalkanoyloxy) Alkanoic Acids and Rhamnolipids by High-Performance Liquid Chromatography-Charged Aerosol Detector

Reversed-phase chromatography and product detection with a charged aerosol detector (CAD) was performed for analysis of HAA, mono- and di-rhamnolipid as described before (Bator et al., 2020). For sample preparation, the cell-free culture broth was mixed 1:1 with acetonitrile and stored at 4°C overnight. Subsequently, the mixture was centrifuged (2 min, 18,000 × g). All samples were filtered with Phenex RC syringe filters (0.2 μm, Ø 4 mm, Phenomenex, Torrance, United States). The HPLC system Ultimate 3000 equipped with a Corona Veo Charged Aerosol Detector (Thermo Fisher Scientific, Waltham, MA, United States) was used with a NUCLEODUR C18 Gravity 150 × 4.6 mm column (particle size: 3 μm, Macherey-Nagel GmbH & Co. KG, Düren, Germany). The flow rate was set to 1 ml/min, and the column oven temperature was set to 40°C. Acetonitrile (A) and 0.2% (v/v) formic acid in ultra-pure water (B) were used as running buffers. The method started with a ratio of A:B from 70:30%, and a linear gradient was applied to reach a ratio of 80:20% in 8 min. The acetonitrile fraction was increased linearly from 80 to 100% between 9 and 10 min and decreased linearly to 70% between 11 and 12.5 min. The measurement was stopped after 15 min.

For preparation of biosurfactant standards, recombinant *P. putida* strains producing HAA, mRL, and dRL were used. The production and purification of the respective C_10_-C_10_ congeners were performed as described previously (Blesken et al., 2020).

### Victoria Pure Blue BO Assay

MTPs containing VPBO coatings were prepared using a 0.1 mg/ml solution of VPBO (Santa Cruz Biotechnology, California, United States) in isopropanol according to a protocol described previously (Roosloot and Schoen, 2011). Each well in a 96-well plate (Microplate, Greiner Bio-One, Kremsmünster, Austria, flat bottom, polystyrene) was filled with 50 μl of VPBO-solution, and the isopropanol was evaporated under vacuum (Speed-Vac concentrator 5301, Eppendorf, Hamburg, Germany, desiccator function, 60 min, 45°C). Afterward, 0.5 M NaOH (300 μl per well) was added and incubated for 10 min at room temperature. The NaOH solution was aspirated, and the plate was dried again (Speed-Vac concentrator 5301, Eppendorf, desiccator function, 60 min, 45°C). After this stage, the ready-to-use plates were used directly or stored at 4°C sealed with an aluminum foil plate seal.

For the reference measurements, serial dilutions of pure biosurfactants (C_10_-C_10_ congeners of HAA, mRL, and dRL, as well as *N*-myristoyltyrosine) were prepared in LB medium. Specifically, 18–30 dilutions were prepared for each surfactant in the following concentration ranges: between 0.01 and 5 mg/ml for HAA, between 0.01 and 3.6 mg/ml for mRL, between 0.01 and 5 mg/ml for dRL, and between 0.01 and 2.25 mg/ml for *N*-myristoyltyrosine (here from a stock solution with 2 mM NaOH). Aliquots of each dilution (250 μl/well) were transferred to the plate in triplicates. For biological samples, likewise, 250 μl of culture supernatant (30 s, 18,000 × g) was transferred in triplicates to the assay plate. The plate was sealed with an aluminum plate seal and incubated for 1 h at 23°C and 750 rpm (Thermomixer C, Eppendorf, Hamburg, Germany). Afterward, aliquots of 200 μl were transferred to a clean 96-well microplate, and the VPBO-dependent absorbance of the solution was determined at a wavelength of 625 nm (infinite M1000 Pro fluorescence microplate reader, Tecan). For calculation of biosurfactant concentrations in supernatants from the absorption values, logarithmic trendline equations were calculated *via* Excel (Microsoft, Redmond, US) from the plots for the pure biosurfactant serial dilution. Verduyn or LB medium was used as negative control, 100 mM Tween 80 solution as positive control for the assay.

Changes of VBPO absorption at different pH values were determined with LB medium adjusted to pH 2 or 13 with HCl and NaOH, respectively. The absorption spectra were recorded using a spectrophotometer (Genesys 10S UV-Vis, Thermo Fisher Scientific, Waltham, United States) with a quartz cuvette (SUPRASIL CG, 10 × 2 mm). In addition, serial dilutions of the commonly used detergents cetyltrimethylammonium bromide (CTAB, Carl Roth), Tween 80 (Carl Roth), and sodium dodecyl sulfate (SDS, Carl Roth) were prepared in LB medium at concentrations ranging from 0.001 to 5 mg/ml for Tween 80 and CTAB, and 0.0125–5 mg/ml for SDS. The solutions were adjusted to pH 4, 7, and 9 and analyzed with the VPBO assay as described above.

## Results

### Victoria Pure Blue BO Assay for Determination of Biosurfactants

**FIGURE 1:**
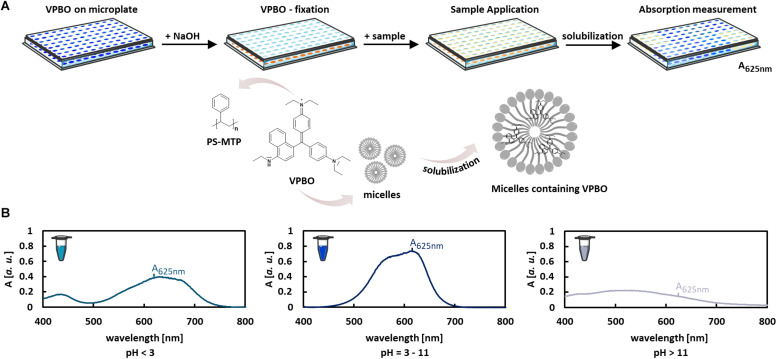
Colorimetric VPBO biosurfactant assay. **(A)** Illustration of the workflow: VPBO is immobilized in the wells of a polystyrene MTP after treatment with NaOH. For the detection of surface-active ingredients, samples are added, leading to solubilization of the blue dye. This can be subsequently detected by absorption measurement at 625 nm after transfer of the liquid to a fresh MTP. Pictures of MTP and reaction tubes were retrieved from Servier Medical Art (https://smart.servier.com/) licensed under Creative Commons Attribution 3.0 (CC BY). **(B)** The pH capacity of the VPBO dye as shown by the absorption behavior in solutions of different pHs. A, absorption; VPBO, Victoria Pure Blue BO; PS, polystyrene; MTP, microtiter plate.

Several microbial species produce biosurfactants and secrete them into the culture supernatant. Usually, extractions are required as a first step for the determination of biosurfactant amounts and titers. Here, we have adapted a method utilizing surfactant-dependent dye solubilization to quantify detergents in vaccine preparations (Roosloot and Schoen, 2011) for the quantification of biosurfactants in culture supernatants using rhamnolipids as model compounds. In contrast to, e.g., orcinol or anthrone reactions for rhamnolipid detection, this method relies on the surface activity of compound, not on a less specific chemical reaction with reducing sugars in general. In contrast to drop collapsing and emulsification assays, this method results in quantitative data and additionally enables higher throughput than current colorimetric semiquantitative or HPLC-based methods. The experimental strategy is schematically illustrated in Figure 1. VPBO is dried in 96-well plates and treated with NaOH, which leads to a discoloration (Figure 1A) and immobilization of the dye on the polystyrene surface. Dried plates containing VPBO can be readily used for sample application. Solubilization of the immobilized dye by a (bio)surfactant results in an increase of the VPBO-specific absorption by the (bio)surfactant preparation in buffer or the surfactant-containing culture supernatant (Roosloot and Schoen, 2011). The amount of released dye can be quantified spectrophotometrically at 625 nm after the transfer of the sample to a clean MTP. Complete solubilization of the fixed VPBO (attached to the plastic wall of the MTP wells) was achieved with 250 μl of a 100-mM Tween 80 solution as described by Roosloot and Schoen (2011) and leads to an absorption value of around 1.1 in our setup. The VPBO assay appears rather pH robust as the dye shows a stable absorption spectrum at pH values in the range of pH 3–11 (Figure 1B). We also determined with the VPBO solubilization by the synthetic surfactants Tween 80, CTAB, and SDS at pH 4, 7, and 9, indicating that the absorption remained nearly constant (Supplementary Figure S1). Nevertheless, as the assembly behavior of biosurfactants to macrostructures typically changes with pH (Jahan et al., 2020), it is important to work in a pH range that allows the formation of solubilizing biosurfactant micelles.

### Quantification of Biosurfactants

**FIGURE 2:**
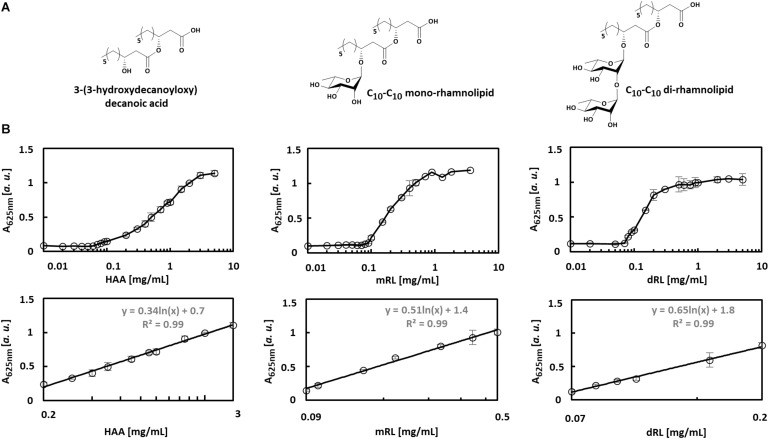
Concentration-dependent solubilization of VPBO by different biosurfactants. **(A)** Chemical structures of biosurfactants C_10_-C_10_-HAA, C_10_-C_10_-mRL, C_10_-C_10_-dRL. **(B)** Biosurfactants were tested at different concentrations plotted in log scale on the x-axis, and the absorbance of VPBO released was measured at 625 nm and plotted on the y-axis (upper row). Concentrations and signal intensities were correlated to obtain calibration curves for each compound in the concentration range in which the logarithmically plotted slope is linear and *R*^2^ > 0.99. The respective sections of the plots are depicted (lower row). The respective logarithmic equations usable for surfactant quantification are: for HAA: y = 0.34ln(x) + 0.7 in a range from 0.2 to 3 mg/ml, for mRL: y = 0.51ln(x) + 1.4 for a range from 0.09 to 0.5 mg/ml, for dRL: y = 0.65ln(x) + 1.8 for a range from 0.07 to 0.2 mg/ml. A, absorption; HAA, = 3-(3-hydroxydecanoyloxy) decanoic acid; mRL, C_10_-C_10_ mono-rhamnolipid; dRL, C_10_-C_10_ di-rhamnolipid; VPBO, Victoria Pure Blue BO.

The applicability of the VPBO assay for quantification of biosurfactants in culture medium was tested with HAA, mRL, and diRL, which are structurally and physicochemically different compounds (Figure 2A) produced and secreted by engineered *P. putida* (Tiso et al., 2017). We prepared serial dilutions of C_10_-C_10_ congeners of HAA, mRL, and dRL in sterile LB medium to mimic application conditions and performed the VPBO assay. In all cases, a concentration-dependent increase of absorbance following a sigmoidal curve was observed in a logarithmic plot (Figure 2B). For HAA, the curve showed a relatively flat slope. The absorption starts to increase at an HAA concentration of about 0.1 mg/ml and reaches the maximal absorption marking the solubilization of the entire dye at about 3 mg/ml. In the case of mRL, the slope is steeper, and the solubilization of dye by this surfactant appears to be more effective. It starts at around 0.08 mg/ml and reaches a maximum of absorbance at a concentration of 0.9 mg/ml. Application of dRL-solutions led to an even steeper slope starting at a concentration of 0.06 mg/ml and ending at 0.2 mg/ml. The differential behavior of the three rhamnolipid surfactants may reflect the differences in the solubilization efficiency for hydrophobic compounds. The concentration at which solubilization starts can be considered as the CMC.

The clearly concentration-dependent increase of absorbance of the LB-biosurfactant-solution should enable the quantification of those metabolites in complex environments like culture supernatants. Therefore, absorbance data points in the slope areas were fitted using a logarithmic trend. This approach yielded calibration curves fulfilling a *R*^2^ > 0.99 within a range of 0.2–3 mg/ml for HAA, 0.09–0.5 mg/ml for mRL, and 0.07–0.2 mg/ml for dRL.

### Quantification of Rhamnolipid Biosurfactants in Culture Supernatants

Rhamnolipids produced by different bacteria are commonly a mixture of certain congeners differing, for example, in the length of the fatty acid chains (Abdel-Mawgoud et al., 2010). We used the VBPO assay to quantify different rhamnolipid-derived biosurfactants using recombinant *P. putida* KT2440 harboring plasmid pSB01 with the gene PA_*rhlA*, pPS05 with the genes PA_*rhlAB*, and pWJ02 with the genes PA*_rhlABC* for the production of HAA, mRL, and dRL, respectively. These strains were cultivated for 24 h, and the production of rhamnolipid-derived biosurfactants was confirmed by HPLC-CAD analysis (Supplementary Table S1). The analysis showed that the supernatants contained the typical distribution of congeners with C_8_-C_10_, C_10_-C_10_, C_10_-C_12:1_, and C_10_-C_12_ fatty acids that usually occur during heterologous production of HAA, mRL, or dRL upon expression of *P. aeruginosa rhl* genes in *P. putida* KT2440 (Behrens et al., 2016). Besides, the analysis revealed that *P. putida* KT2440 pJW02 produced dRL with a minor fraction of mRL (ca. 20% of total RL after 24 h). This was expected since exclusive dRL synthesis apparently does not occur in biological production systems.

**FIGURE 3:**
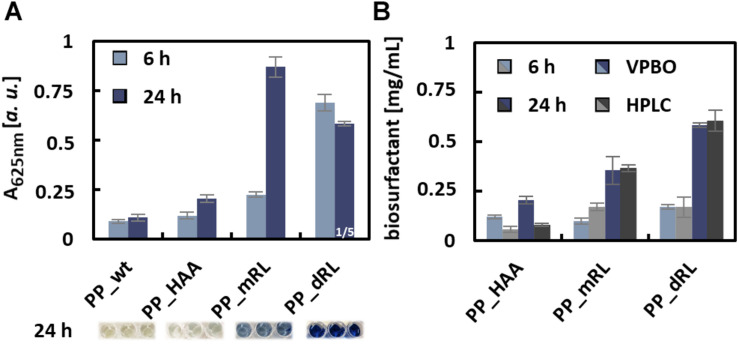
Quantification of rhamnolipid biosurfactants in culture supernatants with the VPBO assay. **(A)** Absorption values at 625 nm obtained with the culture supernatants, sampled after 6 and 24 h of cultivation from PP_wt, PP_HAA, PP_mRL, and PP_dRL. For PP_dRL, the absorption of 1:4 diluted culture supernatant is shown. The dilution in LB medium was necessary to obtain an absorption value within the calibration range. For illustration, the colors of undiluted culture supernatants after the assay are shown below the diagrams. Data represent mean values with standard deviations of biological and technical triplicates. **(B)** Comparison of data obtained from the VPBO assay (blue-shaded bars) with data obtained by HPLC-CAD measurements (gray-shaded bars). Cell-free supernatants of strains PP_HAA, PP_mRL, and PP_dRL were tested after 6 and 24 h of cultivation. A, absorption; VPBO, Victoria Pure Blue BO; PP_wt, *P. putida* KT2440; PP_HAA, *P. putida* KT2440 pSB01; PP_mRL, *P. putida* KT2440 pPS05; PP_dRL, *P. putida* KT2440 pJW02; HPLC-CAD, high-performance liquid chromatography-charged aerosol detector.

Samples taken after 6 and 24 h of cultivation of all production cultures were subjected to the VPBO assay. To exclude acidification of the culture broth by organic acid production that would affect solubility and micelle formation of the surfactants, the pH was measured and found to be in the same range (pH 7.2–7.5) as for pure LB used for calibration. The supernatants of the production strains were able to solubilize VPBO to a much greater extent than the control supernatant of *P. putida* KT2440 without plasmid (Figure 3A). For the control strain, the low absorption value of about 0.1, which is in the same range as the control without any surfactants, indicated that *P. putida* KT2440 itself did not release detectable amounts of solubilizing metabolites into the medium. Furthermore, the supernatants sampled after 24 h from the biosurfactant producing strains led to higher solubilization of the dye than samples taken after 6 h. KT2440 pJW02 supernatants led to an absorption that indicated a dRL titer beyond the concentration that is sufficient to completely solubilize the immobilized dye and therefore out of the range of calibration. Hence, the *P. putida* KT2440 pJW02 supernatant was diluted 1:4 in LB and assayed again (Figure 3A).

The determined absorbance values at 625 nm were used to calculate the biosurfactant titers based on the calibrations obtained with purified congeners. The titers calculated accordingly were compared to results from HPLC-CAD quantification (Supplementary Table S1) to evaluate the accuracy of the assay. For strain *P. putida* KT2440 pPS05, the calculated total amount of biosurfactants after 24 h matched the titers determined by HPLC-CAD with only small deviations (Figure 3B), despite the presence of several congeners in the supernatants of the production strains as opposed to the reference compounds used for calibration. However, the C_10_-C_10_ congener used for calibration represented the main congener in the biological samples with a proportion of more than 70%. The calculated total surfactant amount for *P. putida* KT2440 pJW02 supernatants (i.e., mRL/dRL mixture) matched HPLC measurements remarkably well if the results obtained from the equations for C_10_-C_10_-mRL and C_10_-C_10_-dRL were weighted according to the proportions of both surfactants determined by HPLC-CAD. Of course, for quantification applications of unknown samples from dRL producer strains, a known and reproducible congener ratio would be a prerequisite. The quantification of RL titers using exclusively the dRL calibration did not work out, and the proportion of mRL in the samples appears to have significantly influenced the dye solubilization properties of the mixture. For the HAA producer strain *P. putida* KT2440 pPS05 after 6 and 24 h of cultivation, the VPBO calculated amounts do not fit well to the HPLC-determined amounts probably because the titers were out of the calibration range (0.2 and 3 mg/ml for HAA and 0.09 and 0.5 mg/ml for mRL) (Figure 2). In general, the *in vivo* studies indicated that the VPBO assay is suited for fast determination of rhamnolipid concentrations in culture supernatants.

### Detection of Biosurfactant Production by Different Microorganisms

**FIGURE 4:**
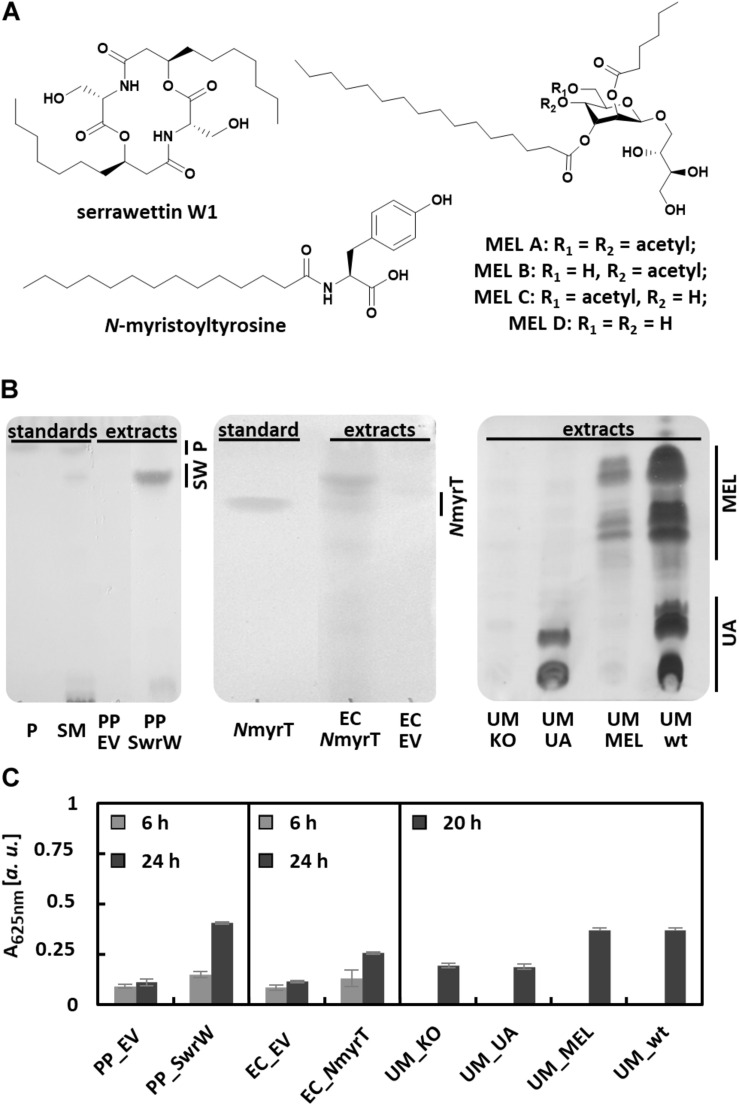
Detection of biosurfactants in culture supernatants with the VPBO assay. **(A)** Chemical structures of tested biosurfactants lipopeptide serrawettin W1, lipoamino acid *N*-myristoyltyrosine, and mannosylerythritol lipids MEL A-D. **(B)** Detection of biosurfactants in supernatant extracts obtained from cultures of the bacterial strains, PP_SwrW, and EC_*N*myrT after 24 h and *U. maydis* strains UM_KO, UM_UA, UM_MEL, and UM_wt after 20 h. Standards for bacterial surfactants consisted of chemically synthesized *N*myrT, extracts from *S. marcescens* DSM12481 containing prodigiosin and serrawettin W1 (SM) and purified prodigiosin (P), the respective running heights are indicated. The strains PP_EV and EC_EV harboring empty vectors served as negative control. Separation was achieved by TLC (Supplementary Figure S3) and subsequent visualization *via* staining with iodine/*p*-anisaldehyde. **(C)** VPBO assay performed with the culture supernatant samples taken from cultures of the bacterial strains after 6 and 24 h cultivation in liquid LB media (measured in biological and technical triplicates) and from cultures of *U. maydis* after 20 h cultivation in liquid modified Verduyn medium (measured in biological duplicates and technical triplicates). VPBO, Victoria Pure Blue BO; TLC, thin-layer chromatography; NmyrT, *N*-myristoyltyrosine; SW, serrawettin W1; IPTG, isopropyl-β-D-thiogalactoside; MEL, mannosylerythritol lipids; UA, ustilagic acids; SM, *S. marcescens* DSM12481; P, prodigiosin; PP_EV, *P. putida* KT2440 pVLT33; PP_SwrW, *P. putida* KT2440 pVLT33-*swrW*; EC_EV, *E. coli* DH10b pEBP18; EC_*N*myrT, *E. coli* DH10b pEBP18_BA354; UM_KO, *U. maydis* MB215 Δcyp1Δemt1; UM_UA, *U. maydis* MB215 Δemt1; UM_MEL, *U. maydis* MB215 Δrua1; UM_wt, *U. maydis* MB215.

The dependence of the VPBO assay on surfactant properties instead of sugar content, charge, or complex formation suggests that this assay may respond to a wide range of surfactants beyond rhamnolipids. We have tested the supernatants of microbes producing different classes of biosurfactants in comparison to non-producing control strains (Figure 4). Addressed compounds included the non-ionic lipopeptide serrawettin W1 (produced with recombinant *P. putida* KT2440), the anionic lipoamino acid surfactant *N*-acyltyrosine produced by recombinant *E. coli* DH10b, and ustilagic acid and the non-ionic MELs (all glycolipids) produced by *U. maydis* MB215 (Figure 4A). Culture supernatants were sampled from bacterial cultures 6 and 24 h after inoculation. For *U. maydis* strains, supernatants were prepared after 20 h of growth. Qualitative analysis using established extraction and TLC protocols for the respective compounds confirmed the production of the biosurfactants (Figure 4B). All supernatants were analyzed with the VPBO assay (Figure 4C). For all surfactant producers, pronounced dye solubilization was indicated by increased absorption at 625 nm except *U. maydis* strain MB215 Δ*emt*1 that produces solely ustilagic acid. The missing response to ustilagic acid probably results from its low solubility in aqueous solution (Lemieux et al., 1951). Hence, the determined absorption obtained with supernatants from the wild-type strain is probably caused by MELs only.

Chemically synthesized *N*-myristoyltyrosine (Thies et al., 2016) was used as a reference to determine the concentration-dependent solubilization of VPBO using a serial dilution in the concentration range of 0.01–2.25 mg/ml. Here, again a sigmoidal curve was obtained, which allowed quantification of the biosurfactant in a concentration range between 0.05 and 1 mg/ml (Supplementary Figure S2). The results showed that the VPBO assay is suitable to detect different types of biosurfactants regardless of cultured microorganisms in different media.

### Assessment of Optimal Inducer Concentration for Biosurfactant Production

**FIGURE 5:**
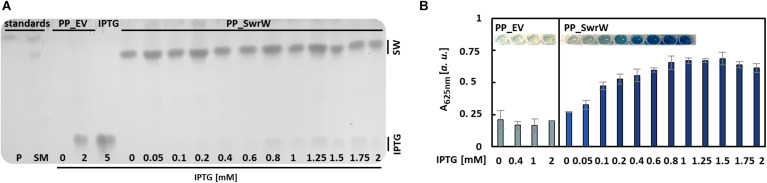
Production of serrawettin W1 with *P. putida* KT2440 containing plasmid pVLT33_*swrW*. Expression of the gene *swrW* was induced by addition of IPTG at different concentrations. **(A)** Iodine stained TLC (Supplementary Figure S3) of ethyl acetate extracts obtained from culture supernatants obtained from cultures of *P. putida* KT2440 harboring plasmids pVLT33 or pVLT_*swrW* and induced with different concentrations of IPTG. Extracts from *S. marcescens* DSM12481 grown on solid tryptone-glycerol medium (SM) were analyzed for comparison to identify spots of prodigiosin (P) and serrawettin W1 (SW). **(B)** VPBO assay with cell-free culture supernatant obtained from *P. putida* KT2440 containing pVLT33 and pVLT_*swrW* after 24 h cultivation in liquid LB medium. Shown are mean values and standard deviations of biological triplicates. A, absorption; VPBO, Victoria Pure Blue BO; TLC, thin-layer chromatography; SW, serrawettin W1; SM, *S. marcescens* DSM12481; P, prodigiosin; IPTG, isopropyl-β-D-thiogalactoside; PP_EV, *P. putida* KT2440 pVLT33; PP_SwrW, *P. putida* KT2440 pVLT33-*swrW*.

Straightforward quantification methods for biosurfactants are particularly useful for screening purposes, e.g., for testing multiple different cultivation conditions, different concentrations of inducers, or identification of efficient producer strains within (meta)genomic libraries or strain collections. As an example, we have tested the production of the biosurfactant serrawettin W1 in 48 parallel cultivations of the expression strain *P. putida* KT2440 harboring plasmid pVLT_*swrW* to identify the optimal concentration of the inducer IPTG, which was supplemented 3.5 h post inoculation to final concentrations between 0 and 2 mM. IPTG induces the expression of the gene *swrW*, which is controlled by the LacI/P*tac* promoter in this vector. After 24 h, the VPBO solubilization potential of the culture supernatants was investigated. The presence of serrawettin W1 in the supernatants of *P. putida* KT2440 pVLT33_*swrW* cultures was confirmed qualitatively by TLC of ethyl acetate extracts. The lipopeptides were visualized by exposure to iodine vapor (Figure 5A). The VPBO assay revealed a low basal production of the non-supplemented strain probably caused by leakiness of the LacI-dependent expression system. Furthermore, we observed increasing concentrations of solubilized dye with rising IPTG concentrations, which indicated a higher concentration of the surfactant in the supernatant up to an IPTG concentration of 0.8 mM, which presumably marks the point of optimal induction or the maximal achievable product accumulation under these conditions (Figure 5B). The complete solubilization of the dye, which would lead to an absorption of 1.1, is apparently not achieved. IPTG did not influence the solubilization indicated by the empty vector control with different IPTG concentrations.

## Discussion

The development of production strategies for biosurfactants such as rhamnolipids gains increasing attention because of their great potential to replace petroleum-based and chemically synthesized surfactants. Therefore, methods are needed to assess product titers easily and rapidly and to compare different production strains. In this study, a sensitive colorimetric assay was developed, which is suitable for time- and resource-efficient screening and identification of biosurfactant producers as well as for biosurfactant quantification if suitable reference compounds are available. It is based on VPBO immobilized on polystyrene MTPs. Presumably, VPBO is deprotonated under basic conditions and attaches by hydrophobic interactions or *via* π-π interactions to the polystyrene surface of a 96-well MTP. If surfactant molecules are present in sufficient concentration, they will form micelles, which results in solubilization of the dye by weakening or even disturbing these interactions.

Compared to other high-throughput assays to determine especially rhamnolipid levels like fluorescein encapsulating vesicles (Laabei et al., 2014; Yang et al., 2015), the VPBO assay is easy to prepare and “ready-to-use” plates can be stored. Additionally, its major component VPBO is readily available and inexpensive. Other colorimetric assays using bromothymol blue (BTB) or cetylpyridinium chloride (CPC), which were described for high-throughput assessment of surfactin titers (Yang et al., 2015), or orcinol reactions for glycolipids (Chandrasekaran and Bemiller, 1980) rely on complex formation, molecular charge, or detection reactions with certain building blocks, which limit their applicability. Often, they require furthermore extraction steps for applications in liquid media, like the cetyltrimethylammonium bromide (CTAB)-methylene blue assay (Pinzon and Ju, 2009). In contrast, the VPBO assay detects the surfactant properties of different compounds directly *via* their ability to solubilize the dye from a plastic surface. Hence, the VPBO assay is insensitive regarding interference of metabolites produced by the microorganisms, e.g., resulting from glucose metabolism as reported for CPC-based methods (Heuson et al., 2019). Notably, compounds with emulsifying properties like polysaccharides coproduced with a biosurfactant of interest may interfere with the assay because it hardly distinguishes between different surfactants. Also, it does not provide direct information about the type of surfactant.

The suitability of the VPBO assay for the fast quantification of biosurfactant concentrations in culture supernatants as shown here for rhamnolipids is in line with reports on accurate quantification of detergents in defined vaccine preparations using a similar assay using dye extracted from ink pens (Roosloot and Schoen, 2011). A direct comparison of the VPBO assay with a vesicle lysis assay failed because this assay did not yield consistent results in our hands. The vesicle lysis assay was described for high-throughput rhamnolipid detection relying on the release of fluorescein from phospholipid vesicles in *P. aeruginosa* culture supernatants (Laabei et al., 2014).

Surfactant-dependent solubilization of extracted ink pen dye was initially described as a straightforward tool to determine the CMC of chemically synthesized surfactants (Vulliez-Le Normand and Eisele, 1993) because dye solubilization starts at a detergent concentration around the CMC (Roosloot and Schoen, 2011). We confirmed these observations for the VBPO assay by measuring absorptions with three synthetic surfactants at three different pH values (Supplementary Figure S1). This suggests that the VPBO assay may be feasible for the characterization of biosurfactants beyond the mere evaluation of concentrations. The CMC values of the four biosurfactants derived from the solubilization curves (Figure 2 and Supplementary Figure S2) were for HAA 0.15 ± 0.05 mg/ml, for mRL 0.09 ± 0.01 mg/ml, for dRL 0.06 ± 0.01 mg/ml, and for *N*-myristoyltyrosine 0.05 ± 0.01 mg/ml. Previous studies report CMC values for dianionic *N*-myristoyltyrosine at pH 12 of about 0.06 mg/ml (Thies et al., 2016), for mRL of 0.04 mg/ml (specifically C_10_-C_10_ congeners), and for dRL (also specifically C_10_-C_10_ congeners) of about 0.005 mg/ml (Rahman et al., 2002). For HAA, a CMC of 0.113 mg/ml was reported for a purified mixture of heterologously produced HAA (Tiso et al., 2017). Hence, for *N*-acyltyrosine, HAA, and mRL, the CMC derived from VPBO assay matches the range of previously reported values, however, for C_10_-C_10_ dRL, it does not. Nevertheless, other studies determined deviating CMCs for dRL (and for mRL as well) varying between 0.05 and 0.23 mg/ml probably dependent on impurities, mixtures, and variations in chain length and degree of saturation (Syldatk et al., 1985; Abalos et al., 2001; Nitschke et al., 2005; Dubeau et al., 2009; Tiso et al., 2017; Rocha et al., 2020). Furthermore, the CMC value depends on temperature, pH, and salt concentration, which may differ from the conditions in the present study (room temperature in LB medium adjusted to pH 7). A more detailed evaluation of the reliability of CMC determination with VPBO to elucidate the current inconclusive data situation should be addressed in further studies.

The VPBO assay may find applications as a robust assay to detect biosurfactant production regardless of their chemical structure, microbial strain, and culture conditions. This assertion is corroborated by the successful detection of the chemically different biosurfactants HAA (anionic fatty acid), mRL, and dRL (anionic glycolipids), N-acyltyrosine (anionic lipoamino acid), and serrawettin W1 (non-ionic lipopeptide), and MELs (non-ionic glycolipids), which are produced by *E. coli* DH10b, *P. putida* KT2440, and the eukaryotic fungus *U. maydis* MB215, respectively. Our observation that ustilagic acid production was not detectable with the VPBO assay indicates that low water solubility of a target biosurfactant may hinder its detection. Hence, assay conditions such as pH should be chosen with care to ensure solubility and the formation of micelles by the surfactants. In our study, rhamnolipid biosurfactants were produced by *P. putida* in a culture broth with a pH suitable to keep them in solution. Hence, it is recommended to adjust the pH or mix the supernatant with a buffer (as suggested by Roosloot and Schoen, 2011) prior to performing the assay. The VPBO assay is also suitable for high-throughput applications where large numbers of clones need to be analyzed in parallel for biosurfactant production as indicated by our experiments to identify the optimal concentration of IPTG for inducing the heterologous production of serrawettin W1 by *P. putida* KT2440. Combining the VPBO assay with other detection methods such as blood agar plates or the atomized oil assay in a screening workflow is feasible as well; such a combination of these assays will certainly increase the chances to detect biosurfactants with physicochemically different properties.

In summary, we have demonstrated that the VPBO assay allows detecting biosurfactant production under a wide range of different conditions. This assay thus constitutes a robust, fast, and inexpensive tool for the detection and, at least in some cases, quantification of different biosurfactants in microbial culture supernatants.

## Data Availability Statement

All datasets generated for this study are included in the article/Supplementary Material.

## Author Contributions

SK and ST designed the experiments. SK, IB, and SJ performed the research and analyzed the data. ST and K-EJ conceived the research concept. SK, ST, KS, MF, TT, LB, and K-EJ wrote and reviewed the manuscript. All authors contributed to the article and approved the submitted version.

## Conflict of Interest

LB and TT declared that they are inventors of three related patents. (1) LB, F. Rosenau, S. Wilhelm, A. Wittgens, and TT, “Means and methods for rhamnolipid production” HHU Düsseldorf University, TU Dortmund University, 2013 (WO 2013/041670 A1); (2) LB, B. Küpper, E. M. del Amor Villa, R. Wichmann, and C. Nowacki, “Foam adsorption” TU Dortmund University, 2013 (WO 2013/087674 A1); and (3) LB, TT, and A. Germer, “Extracellular production of designer hydroxyalkanoyloxy alkanoic acids with recombinant bacteria” RWTH Aachen University, 2015 (WO2017006252A1). The remaining authors declare that the research was conducted in the absence of any commercial or financial relationships that could be construed as a potential conflict of interest.
