# Supplementary material for: A Straightforward Assay for Screening and Quantification of Biosurfactants in Microbial Culture Supernatants
Source: Front Bioeng Biotechnol. 2020 Aug 20;8:958. doi: 10.3389/fbioe.2020.00958 (PMC7468441; doi:10.3389/fbioe.2020.00958)
Supplement: Supplementary file 1 [file Data_Sheet_1.pdf]

Supplementary Material

A straightforward assay for screening and quantification of biosurfactants in microbial culture supernatants

Sonja Kubicki[1,5], Isabel Bator[2,5], Silke Jankowski[3,5], Kerstin Schipper[3,5], Till Tiso[2,5], Michael Feldbrügge[3,5], Lars M. Blank[2,5], Stephan Thies[1,5*], Karl-Erich Jaeger[1,4,5*]

[1]Institute of Molecular Enzyme Technology, Heinrich Heine University Düsseldorf, Jülich, Germany,

[2]iAMB - Institute of Applied Microbiology, ABBt - Aachen Biology and Biotechnology, RWTH Aachen University, Aachen, Germany

[3]Institute for Microbiology, Center of Excellence on Plant Sciences, Heinrich Heine University Düsseldorf, Düsseldorf, Germany,

[4]Institute of Bio-and Geosciences IBG 1: Biotechnology, Forschungszentrum Jülich GmbH, Jülich, Germany

[5]Bioeconomy Science Center (BioSC), Forschungszentrum Jülich GmbH, Jülich, Germany

*** Correspondence:**

E-mail: s.thies@fz-juelich.de; Tel. +(49) 2461 613790
karl-erich.jaeger@fz-juelich.de; Tel. +(49) 2461 613716

1 Supplementary Figures and Tables

1.1 Supplementary Figures

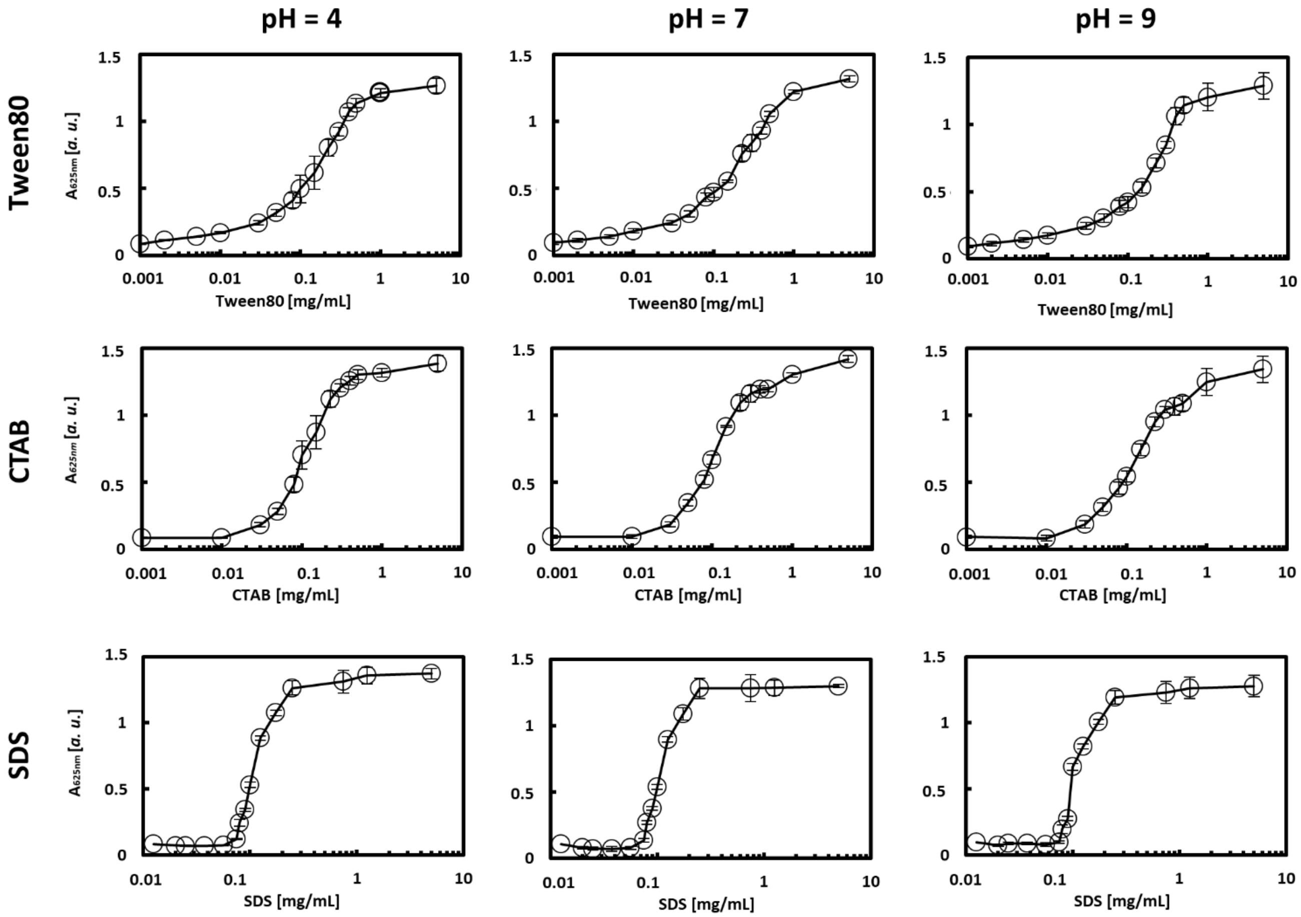

Supplementary Figure 1. **VPBO assay with the synthetic detergents Tween 80, CTAB and SDS in LB medium at different pH-values.**

Serial dilutions of detergents were prepared in LB-medium adjusted to pH 4, 7 and 9 at concentration ranges of 0.001 to 5 mg/mL for Tween 80 (Carl Roth) and cetyltrimethylammonium bromide (CTAB; Carl Roth) and 0.0125 to 5 mg/mL for sodium dodecyl sulfate (SDS; Carl Roth). The CMCs of the surfactants deduced from the absorption curves are 0.02 mg/mL for Tween80, 0.02 mg/mL for CTAB, and 0.08 mg/mL for SDS.

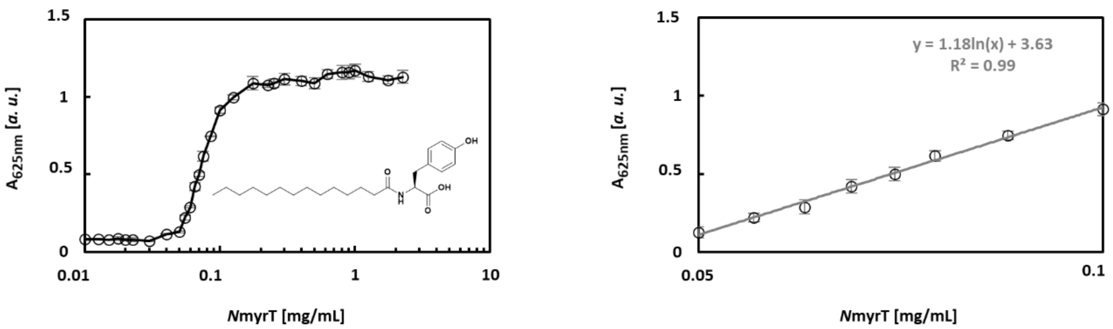

Supplementary Figure 2. **Concentration-dependent solubilization of VPBO by *N*-myristoyltyrosine**.
The absorbance at 625 nm was measured as a function of biosurfactant concentrations and visualized as a log-scaled plot. Linear range of the relation between VPBO absorbance (A_{625}) and concentration of *N*-myristoyltyrosine (mg/mL)**.** The respective logarithmic trendline equation was y = 1.18ln(x) + 3.63 for a range from 0.05 to 0.1 mg/mL.

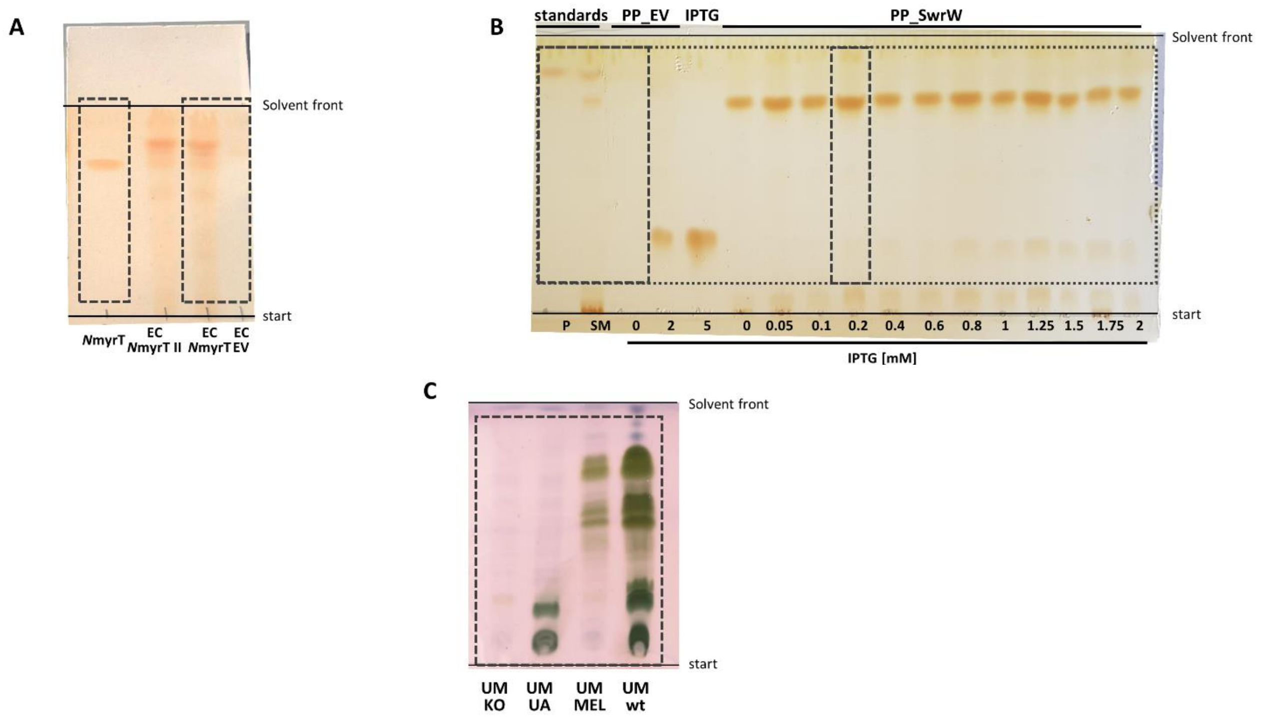

Supplementary Figure 3. Full pictures of the entire TLC plates for detection of biosurfactants in supernatant extracts. This includes supernatants of cultures of EC_*N*myrT (A), PP_SwrW (B) after 24 h and of *U. maydis* strains UM_KO, UM_UA, UM_MEL and UM_wt after 20 h (C). Standards for bacterial surfactants consisted of chemically synthesized *N*myrT, extracts from *S. marcescens* DSM12481 containing prodigiosin and serrawettin W1 (SM) and purified prodigiosin (P). The strains PP_EV and EC_EV harbor empty vectors. Visualization of the biosurfactants was achieved via staining with iodine/ *p*-anisaldehyde. Dashed lines mark the excerpts shown in Figure 4 of the main manuscript; the dotted line in (B) indicates the area shown in Figure 5 of the main manuscript.

1.2 Supplementary Tables

Supplementary Table 1 | Congener titers in supernatants of *P. putida* KT2440 pSB01, pPS05 and pWJ02 determined by HPLC-CAD.

		titer [mg/mL]					
		***P. putida* KT2440 pSB01**		***P. putida* KT2440 pPS05**		***P. putida* KT2440 pWJ02**	
	**congeners**	**6 h**	**24 h**	**6 h**	**24 h**	**6 h**	**24 h**
**HAA**	**C_8-C_{10}**	0.00	0.02	0.00	0.00	0.01	0.00
	**C_{10}-C_{10}**	**0.05**	**0.05**	0.00	0.00	0.01	0.00
	**C_{10}-$C_{12:1}$**	0.00	0.00	0.00	0.00	0.00	0.00
	**C_{10}-C_{12}**	0.00	0.00	0.00	0.00	0.00	0.00
**mRL**	**Rha-C_{10}-C_8**	-	-	0.00	0.02	0.00	0.00
	**Rha-C_{10}-C_{10}**	-	-	**0.06**	**0.24**	0.04	0.08
	**Rha-C_{12}-$C_{10:1}$**	-	-	0.01	0.05	0.01	0.01
	**Rha-C_{12}-C_{10}**	-	-	0.01	0.06	0.01	0.01
**dRL**	**Rha-Rha-C_{10}-C_8**	-	-	-	-	0.00	0.01
	**Rha-Rha-C_{10}-C_{10}**	-	-	-	-	**0.06**	**0.30**
	**Rha-Rha-C_{12}-$C_{10:1}$**	-	-	-	-	0.01	0.08
	**Rha-Rha-C_{12}-C_{10}**	-	-	-	-	0.02	0.10
**∑ total biosurfactant**		**0.06**	**0.08**	**0.08**	**0.37**	**0.17**	**0.60**
